# Factors associated with cytomegalovirus serostatus in young people in England: a cross-sectional study

**DOI:** 10.1186/s12879-020-05572-9

**Published:** 2020-11-23

**Authors:** Joanne R. Winter, Graham S. Taylor, Olivia G. Thomas, Charlotte Jackson, Joanna E. A. Lewis, Helen R. Stagg

**Affiliations:** 1 Centre for Molecular Epidemiology and Translational Research, Institute for Global Health, University College London, London, UK; 2 Institute of Immunology and Immunotherapy, College of Medical and Dental Sciences, University of Birmingham, Birmingham, UK; 3 Present Address: Centre for Molecular Medicine, Karolinska University Hospital Solna, 171 76 Stockholm, Sweden; 4 Present Address: MRC Clinical Trials Unit, University College London, London, UK; 5 National Institute for Health Research (NIHR) Health Protection Research Unit in Modelling Methodology and Medical Research Council Centre for Global Infectious Disease Analysis, School of Public Health, Imperial College London, London, UK; 6 Present Address: Population, Policy and Practice, UCL Great Ormond Street Institute of Child Health, London, UK; 7 Usher Institute, University of Edinburgh, Edinburgh, UK

**Keywords:** Cytomegalovirus, Serostatus, Transmission, Risk factors

## Abstract

**Background:**

Human cytomegalovirus (CMV) is a common herpesvirus which is estimated to infect 83% of the global population. Whilst many infections are asymptomatic, it is an important cause of morbidity and mortality, particularly for immunocompromised people and for infants who are congenitally infected. A vaccine against CMV has been stated as a public health priority, but there are gaps in our understanding of CMV epidemiology. To guide potential future vaccination strategies, our aim was to examine risk factors for CMV seropositivity in young people in England.

**Methods:**

The Health Survey for England (HSE) is an annual, cross-sectional representative survey of households in England during which data are collected through questionnaires, and blood samples are taken. We randomly selected individuals who participated in the HSE 2002, aiming for 25 participants of each sex in each single year age group from 11 to 24 years. Stored samples were tested for CMV antibodies. We undertook descriptive and regression analyses of CMV seroprevalence and risk factors for infection.

**Results:**

Demographic data and serostatus were available for 732 individuals, of whom 175 (23.7%) were CMV-seropositive. CMV seroprevalence was associated with age, with 18.3% seropositive at 11–14 years compared to 28.3% at 22–24 years. CMV serostatus was also higher in people of non-white ethnicity (adjusted odds ratio [aOR] 6.22, 95% confidence interval [CI] 3.47–11.14), and in adults who were seropositive for EBV (aOR 2.08 [1.06–4.09]). There was no evidence that smoking status, occupation, body mass index and region of England were associated with CMV serostatus.

**Conclusions:**

CMV seroprevalence is strongly associated with ethnicity, and modestly increases with age in 11–24-year-olds. A greater understanding of the transmission dynamics of CMV, and the impact of this on CMV-associated morbidity and mortality, is necessary to inform effective vaccination strategies when a vaccine for CMV becomes available.

## Background

The human cytomegalovirus (CMV) is a common human herpesvirus causing lifelong infections, and is estimated to infect 83% of the global population [1]. CMV can be transmitted from symptomatic individuals, via saliva or other body fluids and blood products [2]. Infection with CMV is typically subclinical in healthy individuals [3], however it is linked to multiple causes of morbidity and mortality. CMV accounts for around 5–8% of infectious mononucleosis cases [4, 5], and causes disease in immunocompromised people such as transplant and cancer patients [3]. Congenitally infected newborns can suffer from cytomegalic inclusion syndrome which can cause long-term neurological damage and, in some cases, life-threatening organ dysfunction [5].

CMV infection has been associated with shortened life expectancy, particularly in critically ill populations and immunocompromised people (such as those who have undergone organ transplants) [6]. In immunocompetent people in the UK, Gkrania-Klotsas et al. also found that CMV seropositivity was associated with lower life expectancy [6]. This confirmed the association reported in a population-based cohort study from the US [7], as well as populations in older patients [8] and those with cardiovascular disease [9]. The association found in Gkrania-Klotsas et al. was specifically with deaths from causes other than cardiovascular disease and cancer, although high levels of CMV IgG antibodies were also associated with cardiovascular mortality [6]. Other studies in the United States, Finland and United Kingdom found that, in immunocompetent individuals, CMV infection and higher levels of CMV IgG antibodies were linked to higher rates of both cardiovascular [9] and all-cause mortality [7], as well as to cancer incidence [10] and ischemic heart disease [11]. However, CMV is negatively associated with multiple sclerosis onset [12].

In terms of biological pathways, it has been hypothesised that frequent silent reactivations of CMV infection lead to chronic inflammation, which may be a causal factor in the increased risk of mortality [6]. Additionally, CMV seropositivity has been linked to telomere shortening of T cells, suggesting that CMV may be implicated in immunosenescence, thereby shortening life expectancy [2, 13]. There is also evidence for an immunological phenomenon called ‘memory inflation’, where a high proportion of CD8+ T cells in older CMV-positive individuals react to an epitope from a CMV protein [14]. This may limit the ability of the immune system to respond to other infections and could be associated with CMV’s ability to infect the vascular endothelium. CMV infection also drives large expansions of cytotoxic virus-specific CD4+ T cells in older individuals, which could ‘take up room’ in the immune system and potentially limit responses to other pathogens [15].

Given the implications of CMV infection, anti-CMV vaccines have been designated high priority by national health agencies, but to date no effective vaccines appear to be imminent [16, 17]. Mathematical modelling of the impact of different vaccination strategies can be used to guide vaccine development efforts and will be necessary to inform the optimal strategies for deployment of such a vaccine if or when it becomes available. A thorough understanding of CMV epidemiology is necessary for the development of such models.

CMV seroprevalence increases with age, and infection occurs at younger ages in economically developing countries [2, 6], possibly due to higher rates of breastfeeding than in the UK (CMV is known to be transmitted through breast milk). A large population-based UK cohort study found that CMV infection was more common in women than in men [6]. Lower income and education levels, and ethnicities other than white, have been associated with earlier age at CMV infection [18]. CMV infection is also correlated with EBV infection [19, 20]. Socioeconomic status is strongly correlated with CMV infection; the reasons for this could include larger family size [21], or have been hypothesised to be a result of stress induced by low socioeconomic status contributing to the down-regulation of the immune system and increased susceptibility to infection [18].

To date in the UK, studies of CMV seroprevalence have focused on older adults [6], pregnant women [22], and young children [23, 24]. However transmission can occur at all ages, and the association of CMV with infectious mononucleosis suggests that infection during adolescence could also be an important cause of morbidity. Therefore, our aim was to investigate the sociodemographic and lifestyle factors, particularly age, associated with CMV serostatus in children and young adults in England, in order to gain a better understanding of the epidemiology of CMV in this age group.

## Methods

### Study population

The Health Survey for England (HSE) is a cross-sectional annual representative survey of English households. The methods have been described previously [25]. As part of a larger study investigating Epstein-Barr virus infection and transmission [20, 26], we used data from randomly selected participants in the 2002 HSE, aiming for 25 male and 25 female participants in each single-year age group from 11 to 24 years.

### Outcome: Seropositivity for Cytomegalovirus infection

We used stored blood serum samples collected by the HSE. Commercial ELISA kits from EUROIMMUN, Germany (EI2791–9601-G, EI2570-9601G) were used to detect CMV-specific IgG and EBV viral capsid antigen (VCA)-specific IgG from the serum samples. Assays were conducted according to the manufacturer’s instructions, and we calculated serum antibody concentrations using a standard curve. Results were presented in relative units (RU/mL) using the following thresholds; samples of <16RU/mL were classed as negative, ≤16 to <22RU/mL were borderline, and ≥ 22RU/mL were positive. For the analyses presented here, borderline results (CMV *n* = 1, EBV *n* = 5) were considered seropositive [20].

### Statistical analysis

We used Stata version 15.0 for data analysis. Stata’s *svy* commands were used to weight our sample to be representative of the age and sex of the 2002 English population, using data from the Office for National Statistics [27]. All stated percentages are weighted. We conducted descriptive analyses of the study population. We used ArcMap 10.3.1 to map CMV seroprevalence by English Government Office Region [28].

We used logistic regression models to investigate factors associated with being seropositive for CMV. We used a causal inference framework to identify a priori factors that needed to be included in multivariable models, drawing on the available data from the HSE. This resulted in two multivariable regression models. We built a ‘whole-population’ model, which included our entire study population, to examine the following factors: age, sex, ethnicity (categorised as ‘white’ or ‘other’ due to small numbers of non-white participants), body mass index (BMI; categorised as ‘underweight’ [BMI < 20], ‘healthy weight’ [20–25], ‘overweight’ [25–30] or ‘obese’ [> 30]), region of England, and EBV serostatus. Additionally, we built a second ‘adults-only’ model, which only included participants aged ≥16 years, and additionally included data from questions which were only asked of adults; smoking status (never smoked, current smoker, smoked in past) and occupational category (higher managerial and professional, intermediate occupations, routine and manual occupations, never worked or long-term unemployed, and other). Individuals missing data on one or more variables were excluded from the regression modelling.

### Ethical approval

This study was approved by the University College London Research Ethics Committee (5683/002). The HSE obtained informed written consent from participants at the time of recruitment for blood samples to be collected and stored for future analyses. [15] A parent/guardian of participants also provided written consent for the interviewing of participants who were younger than 16 years, and for the taking of blood samples from participants who were younger than 18 years.

## Results

**Fig. 1:**
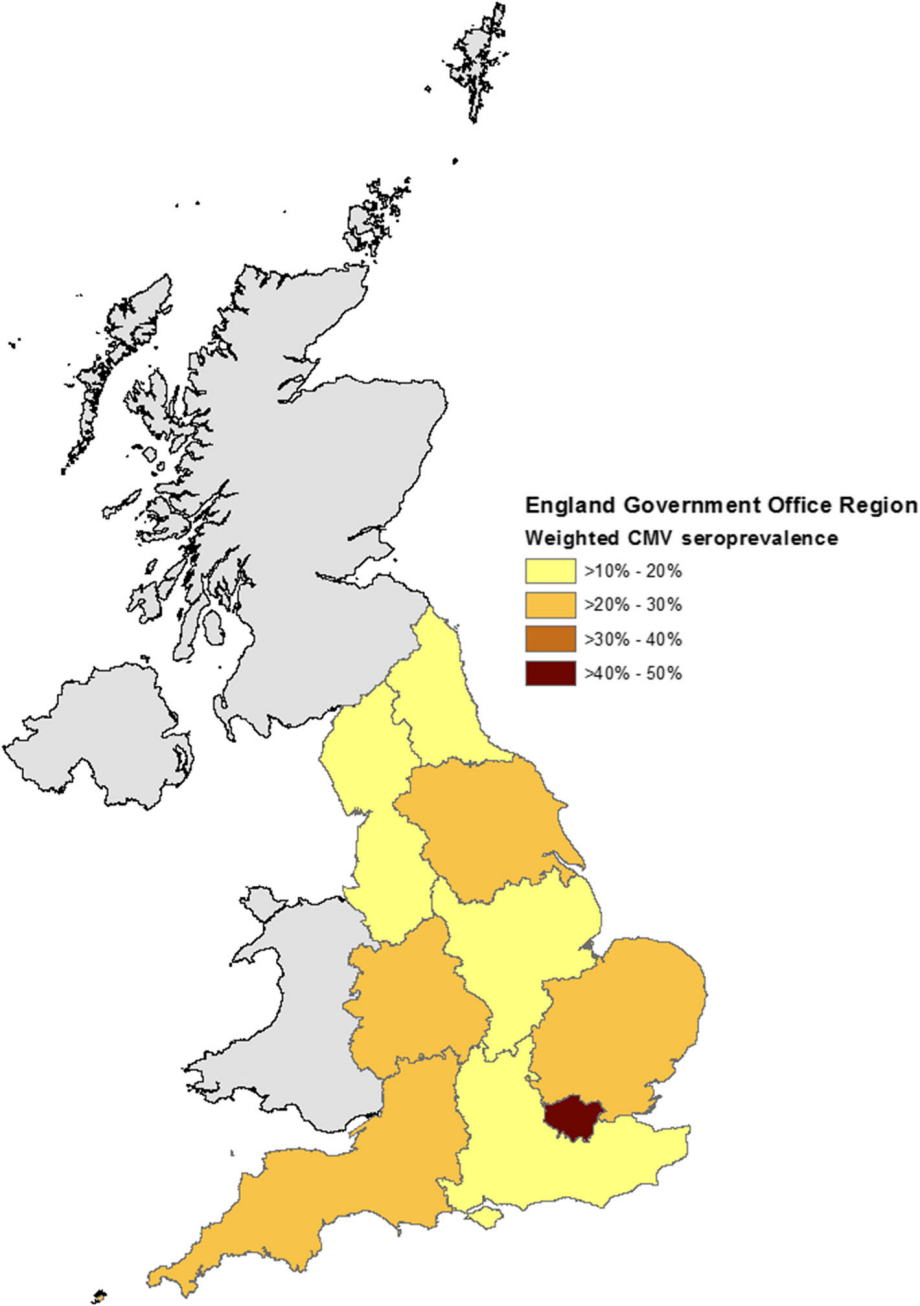
Weighted Cytomegalovirus seroprevalence by English Government Office Region in 2002. Contains National Statistics data©. Crown copyright and database right [2011]. Contains public sector 6 information licensed under the Open Government Licence v3.0

Our study sample included 732 individuals aged 11–24 years, of whom 175 (23.7%) were CMV-seropositive. Seroprevalence by participant characteristics are shown in Table 1. There was a slight increase in CMV seropositivity associated with age, from 18.3% at 11–14 years to 28.3% at 22–24 years. CMV seroprevalence was much lower in white people (19.2%) than people of other ethnicities (61.6%). Considerable variation in CMV seroprevalence was observed by region of England (Fig. 1, Table 1), being highest in London (47.9%) and otherwise varying between 16.7% in the south-east and 28.7% in the east of England. CMV serostatus was also higher in women (26.9%) than in men (20.4%) and people who were EBV-seropositive (25.7%) than EBV-seronegative (17.9%).

**Table 1:** The baseline characteristics of the study population and number and weighted percentage of individuals seropositive for CMV in England in 2002

Variable	Total number	Number CMV seropositive (weighted %)
**Total**	732	175 (23.7)
**Sex**		
Male	364	74 (20.4)
Female	368	101 (26.9)
**Age at last birthday (years)**		
11–14	208	39 (18.3)
15–18	212	52 (24.6)
19–21	156	40 (25.6)
22–24	156	44 (28.3)
**Ethnicity**		
White	665	127 (19.2)
Other	77	48 (61.6)
**BMI**		
Underweight	60	17 (28.1)
Healthy weight	418	94 (22.0)
Overweight	141	34 (23.8)
Obese	87	25 (29.9)
Missing	26	5 (19.4)
**EBV serostatus**		
EBV-seronegative	547	34 (17.9)
EBV-seropositive	185	141 (25.7)
**Region of England**		
East of England	78	24 (28.7)
North East	34	6 (18.0)
North West	130	27 (19.7)
Yorkshire and The Humber	82	18 (22.1)
East Midlands	74	13 (17.0)
West Midlands	70	16 (23.4)
London	63	30 (47.9)
South East	119	20 (16.7)
South West	82	21 (26.4)
**Smoking status**^**a**^		
Never smoked	86	52 (27.9)
Current smoker	134	35 (26.0)
smoker	147	36 (24.5)
Missing	5	3 (60.0)
**Occupational category**^**a**^		
Higher managerial and professional	83	26 (31.6)
Intermediate occupations	69	20 (28.9)
Routine and manual occupations	254	61 (24.0)
Never worked or long-term unemployed	11	2 (18.1)
Other	55	17 (30.3)

^a^Adults aged ≥16 years only (*n* = 472). Percentages account for the weighting of the sample to be representative of the English population in 2002 with respect to age and sex. *BMI* body mass index, *CI* confidence interval, *CMV* cytomegalovirus, *EBV* Epstein-Barr virus

Univariable and multivariable regression models were built examining the factors associated with CMV positivity. Factors associated with CMV seropositivity were largely consistent between the univariable and multivariable models (Table 2), although confidence intervals tended to be wider in the multivariable models. Ethnicities other than white were strongly associated with CMV seropositivity in both univariable and multivariable models (adjusted odds ratio [aOR] 6.22, 95% confidence interval [CI] 3.47–11.14). EBV serostatus was associated with CMV serostatus in the univariable model (odds ratio [OR] 1.59 [1.06–2.39] and in the adults-only multivariable model (aOR 2.08 [1.06–4.09]), but not in the multivariable model which included children (aOR 1.21 [0.76–1.92]). Female sex was associated with higher CMV positivity in the univariable model (OR 1.44, 1.02–2.02); the multivariable models had similar point estimates, but the confidence intervals included unity. Region of England was not associated with CMV serostatus in multivariable models. Neither smoking status nor occupation were associated with CMV serostatus in adults.

**Table 2:** Univariable and multivariable logistic regression models of factors associated with Cytomegalovirus seropositivity in England in 2002

		Whole-population	Adults only^a^
	Univariable OR (95% CI)	Multivariable aOR (95% CI)	Multivariable aOR (95% CI)
**Sex**			
Male	1.00	1.00	1.00
Female	1.44 (1.02–2.02)	1.39 (0.95–2.05)	1.50 (0.95–2.35)
**Age at last birthday (years)**			
11–14	1.00	1.00	
15–18^†^	1.46 (0.89–2.40)	1.55 (0.88–2.75)	1.00
19–21	1.54 (0.89–2.66)	2.24 (1.21–4.14)	1.05 (0.58–1.91)
22–24	1.76 (1.04–2.99)	1.77 (0.95–3.29)	0.74 (0.38–1.46)
**Ethnicity**			
White	1.00	1.00	1.00
Other	6.75 (4.23–10.77)	6.22 (3.47–11.14)	6.98 (3.18–15.32)
BMI			
Underweight	1.39 (0.77–2.52)	1.18 (0.62–2.24)	0.98 (0.50–1.93)
Healthy weight	1.00	1.00	1.00
Overweight	1.11 (0.71–1.74)	1.15 (0.71–1.86)	0.84 (0.47–1.53)
Obese	1.52 (0.90–2.56)	1.50 (0.81–2.77)	1.06 (0.40–2.76)
**EBV serostatus**			
Negative	1.00	1.00	1.00
Positive	1.59 (1.06–2.39)	1.21 (0.76–1.92)	2.08 (1.06–4.09)
**Region of England**			
East of England	1.00	1.00	1.00
North East	0.54 (0.18–1.66)	0.51 (0.17–1.50)	0.57 (0.16–2.02)
North West	0.61 (0.31–1.19)	0.62 (0.31–1.22)	0.60 (0.25–1.40)
Yorkshire and The Humber	0.70 (0.33–1.51)	0.67 (0.30–1.51)	0.62 (0.26–1.47)
East Midlands	0.51 (0.21–1.19)	0.52 (0.21–1.26)	0.70 (0.24–2.04)
West Midlands	0.76 (0.35–1.63)	0.81 (0.36–1.79)	0.53 (0.18–1.60)
London	2.28 (1.13–4.61)	1.18 (0.52–2.69)	1.28 (0.45–3.61)
South East	0.50 (0.24–1.03)	0.43 (0.20–0.93)	0.44 (0.17–1.14)
South West	0.89 (0.46–1.70)	1.09 (0.55–2.15)	0.72 (0.29–1.78)
**Smoking status**^a^			
Never smoked	1.00	–	1.00
Current smoker	0.91 (0.54–1.51)	–	1.08 (0.58–2.01)
Smoked in past	0.84 (0.51–1.38)	–	0.96 (0.54–1.69)
**Occupational category**^a^			
Higher managerial and professional	1.00	–	1.00
Intermediate occupations	0.88 (0.44–1.76)	–	0.66 (0.31–1.42)
Routine and manual occupations	0.68 (0.40–1.18)	–	0.63 (0.32–1.24)
Never worked or long-term unemployed	0.48 (0.09–2.45)	–	0.40 (0.07–2.14)
Other	0.94 (0.46–1.93)		0.47 (0.17–1.28)

^a^Adults aged ≥16 years only (n = 472). ^†^16–18 years for ‘adult-only’ model. Odds ratios account for the weighting of the sample to be representative of the English population in 2002 with respect to age and sex. The ‘whole population’ multivariable model included age, sex, CMV serostatus, ethnicity, BMI and region of England. The ‘adults only’ multivariable model included all variables shown in the table. *aOR* adjusted odds ratio, *BMI* body mass index, *CI* confidence interval, *CMV* cytomegalovirus, *OR* unadjusted odds ratio

Our study sample included 732 individuals aged 11–24 years, of whom 175 (23.7%) were CMV-seropositive. The characteristics of seropositive individuals are shown in Table 1. Univariable and multivariable regression models were built examining the factors associated with CMV positivity. Factors associated with CMV seropositivity were largely consistent between the univariable and multivariable models (Table 2), although confidence intervals tended to be wider in the multivariable models.

There was an increase in CMV seropositivity associated with age, from 18.3% at 11–14 years to 28.3% at 22–24 years, but the confidence intervals between strata overlapped in logistic regression models. CMV seroprevalence was much higher in people of non-white ethnicities than in white people (61.6% vs 19.2%; aOR 6.22, 95% CI 3.47–11.14). CMV serostatus was also higher in women (26.9%) than in men (20.4%); the CI for this association excluded unity in a univariable model (odds ratio [OR] 1.44, 95% confidence interval [CI] 1.02–2.02); the multivariable models had similar point estimates, but the confidence intervals included unity. Neither smoking status nor occupation were associated with CMV serostatus in adults.

CMV seropositivity was higher in people who were EBV-seropositive (25.7%) than EBV-seronegative (17.9%). EBV serostatus was associated with CMV serostatus in the univariable model (OR 1.59 [1.06–2.39] and in the adults-only model (aOR 2.08 [1.06–4.09]), but not in the multivariable model which included children (aOR 1.21 [0.76–1.92]).

Considerable variation in CMV seroprevalence was observed by region of England (Fig. 1, Table 1), CMV seroprevalence was highest in London (47.9%) and otherwise varied between 16.7% in the south-east to 28.7% in the east of England. However, region of England was not associated with CMV serostatus in multivariable models.

## Discussion

In this study of young people in England, we found that just under a quarter of people aged 11–24 years were infected with CMV, and that seroprevalence increased over this age range. CMV infection was also strongly correlated with non-white ethnicity and more weakly associated with EBV infection. There was no association observed between CMV and region of England, smoking status, BMI, or occupation.

CMV and EBV serostatus were positively associated in univariable analyses, and when the multivariable analysis was restricted to adults, but not in the multivariable model which also included children aged 11–15 years. As discussed in our previous paper [20], both CMV and EBV are associated with increasing age, however EBV increases more rapidly during adolescence than CMV. Thus, in a whole-cohort model adjusting for age, this association may not be visible. As the associations between age and both CMV and EBV [20] are less strong in adults, it is possible that there was enough of a residual effect that the association between CMV and EBV could be detected in the adults-only model. Given the cross-sectional nature of our study, the relative temporality of the two infections could not be assessed. Although established, the relationship between CMV and EBV is not well understood. It is known that EBV seroprevalence is higher than CMV seroprevalence in all age groups and that both increase with age [20], but it is not known whether this relationship is causal or whether the association results from shared genetic, immunological and/or sociodemographic risk factors. Longitudinal studies with serial testing and a larger sample size would be necessary to explore this association in more detail.

We observed a strong association between ethnicity and CMV seroprevalence; the odds of being CMV positive were approximately seven time higher for people of ethnicities other than white than for white people. This may be the result of different social mixing patterns, larger households, different eating or hygiene habits, lower breastfeeding rates in white people (resulting in less vertical transmission of CMV through breastmilk), possibly different countries of birth (of participants or their parents) or residual confounding of socioeconomic status. This strong association with ethnicity is also likely to be a confounder in the association between CMV and region, particularly London, that was only observed in univariable models, as there is a higher proportion of ethnic minorities living in London than elsewhere in England [29]. We were unable to analyse associations with ethnicity in more detail due to small numbers of participants; the “non-white” group comprised 57% Asian/Asian British (*n* = 44), 19% black/black British (*n* = 15), 14% mixed ethnicity (*n* = 11) and 9% other ethnicity (*n* = 7). Further study of the association of ethnicity with CMV seroprevalence is needed in diverse cohorts.

Our study benefits from a sample drawn from a highly rigorous, annual, representative survey of people in England, which we weighted to be representative of the English population, and the use of a quality-managed commercial assay to measure the antibody response. The limitations of our work include the use of a cross-sectional study design, preventing determination of the temporality of certain associations, and the age of the data; 2002 was the most recent year for which the HSE collected consent to analyse blood samples for blood-borne viruses. More recent data from the UK biobank found that 58% of those aged 40–69 years were seropositive for CMV at enrolment (2006–2010) [30], and as CMV seroprevalence increases with age throughout life, the prevalence observed in young people in our study is consistent with what could be expected. An older study examined CMV seroprevalence in 1991 and 2002 and found that prevalence in young people did not differ between these two timepoints [31], and so there is no particular reason to believe CMV seroprevalence has changed substantially since then. We also consider it unlikely that the associations between CMV and the risk factors we studied would have changed substantially since 2002, and therefore the associations we observed are likely to be consistent today even if there had been a slight change in CMV seroprevalence.

The relatively low seroprevalence of CMV meant this study may have lacked power to detect associations, particularly in the multivariable models. We were also limited in the variables that were available, we were unfortunately unable to examine associations with household size or household income. Additionally, the geographical variables available were lacking in granularity, meaning we were not able to explore regional differences in more depth or examine whether regional variation was associated with other sociodemographic risk factors.

We observed only a modest increase in CMV seroprevalence associated with age, suggesting that adolescence is not a key transmission period for CMV as it is for EBV (for which seroprevalence increases from 60% in 11–14 year olds to 93% in 22–24 year olds [20]). This may contribute to the lower incidence of CMV-associated (versus EBV-associated) infectious mononucleosis [4, 5]. Previous studies have shown that 15% of white British and 44% of British Pakistani infants were infected with CMV by the age of 2 years, and that seroprevalence was 59% in an adult cohort aged 40–79 years. In combination with our results, this suggests that after early childhood, there is no ‘key’ age group in which CMV seroprevalence sharply increases, and that infection continues to increase during adulthood, particularly for white British individuals. A better understanding of the interactions between age at CMV infection, and the development of CMV-related morbidity and mortality, is necessary to be able to develop an appropriate vaccination strategy, when a vaccine becomes available.

## Conclusions

CMV seroprevalence is strongly associated with ethnicity, and modestly increases with age. A greater understanding of the transmission dynamics of CMV, and the impact of this on CMV-associated morbidity and mortality, is necessary to inform effective vaccination strategies when a vaccine for CMV becomes available.

## Data Availability

The data used in this study was under license from the Health Survey for England, and so are not publicly available, but can be requested from the HSE.

## Abbreviations

aOR: adjusted odds ratio
BMI: Body mass index
CI: Confidence interval
CMV: Cytomegalovirus
EBV: Epstein-Barr virus
HSE: Health survey for England
OR: Odds ratio
RU: Relative units
UK: United Kingdom
VCA: Viral capsid antigen

## References

[1] Zuhair M (2019). Estimation of the worldwide seroprevalence of cytomegalovirus: a systematic review and meta-analysis. Rev Med Virol.

[2] Arens R, Remmerswaal EBM, Bosch JA, van Lier RAW (2015). 5th international workshop on CMV and Immunosenescence - a shadow of cytomegalovirus infection on immunological memory. Eur J Immunol.

[3] Navarro D (2016). Expanding role of cytomegalovirus as a human pathogen: Cytomegalovirus and human disease. J Med Virol.

[4] Evans A (1978). Infectious mononucleosis and related syndromes. Am J Med Sci.

[5] Landolfo S, Gariglio M, Gribaudo G, Lembo D (2003). The human cytomegalovirus. Pharmacol Ther.

[6] Gkrania-Klotsas E (2013). Seropositivity and higher immunoglobulin G antibody levels against Cytomegalovirus are associated with mortality in the population-based European prospective investigation of Cancer–Norfolk cohort. Clin Infect Dis.

[7] Simanek AM (2011). Seropositivity to cytomegalovirus, inflammation, all-cause and cardiovascular disease-related mortality in the United States. Plos One.

[8] Wang GC (2010). Cytomegalovirus infection and the risk of mortality and frailty in older women: a prospective observational cohort study. Am J Epidemiol.

[9] Strandberg TE, Pitkala KH, Tilvis RS (2009). Cytomegalovirus antibody level and mortality among community-dwelling older adults with stable cardiovascular disease. JAMA.

[10] Lepiller Q, Tripathy MK, Di Martino V, Kantelip B, Herbein G (2011). Increased HCMV seroprevalence in patients with hepatocellular carcinoma. Virol J.

[11] Gkrania-Klotsas E (2012). Higher immunoglobulin G antibody levels against Cytomegalovirus are associated with incident ischemic heart disease in the population-based EPIC-Norfolk cohort. J Infect Dis.

[12] Sundqvist E (2014). Cytomegalovirus seropositivity is negatively associated with multiple sclerosis. Mult Scler Houndmills Basingstoke Engl.

[13] van de Berg PJEJ (2010). Cytomegalovirus infection reduces telomere length of the circulating T cell pool. J Immunol Baltim Md.

[14] Hosie L, et al. Cytomegalovirus-specific T cells restricted by HLA-Cw*0702 increase markedly with age and dominate the CD8+ T-cell repertoire in older people. Front Immunol. 2017;8:1776. 10.3389/fimmu.2017.01776 PMC5732243 29312307

[15] Pachnio A (2016). Cytomegalovirus infection leads to development of high frequencies of cytotoxic virus-specific CD4+ T cells targeted to vascular endothelium. PLoS Pathog.

[16] Sung H, Schleiss MR (2010). Update on the current status of cytomegalovirus vaccines. Expert Rev Vaccines.

[17] Modlin JF (2004). Vaccine development to prevent Cytomegalovirus disease: report from the National Vaccine Advisory Committee. Clin Infect Dis.

[18] Dowd JB, Aiello AE, Alley DE (2009). Socioeconomic Disparities in the Seroprevalence of Cytomegalovirus Infection in the U.S. Population: NHANES III. Epidemiol Infect.

[19] Levine H (2012). Seroepidemiology of Epstein-Barr virus and cytomegalovirus among Israeli male young adults. Ann Epidemiol.

[20] Winter JR (2019). Predictors of Epstein-Barr virus serostatus in young people in England. BMC Infect Dis.

[21] Lachmann R, et al. Cytomegalovirus (CMV) seroprevalence in the adult population of Germany. Plos One. 2018;13:e0200267. 10.1371/journal.pone.0200267 PMC6059406 30044826

[22] Pembrey L, et al. Seroprevalence of Cytomegalovirus, Epstein Barr Virus and Varicella Zoster Virus among Pregnant Women in Bradford: A Cohort Study. Plos One. 2013;8:e81881. 10.1371/journal.pone.0081881 PMC3842274 24312372

[23] Pembrey L, et al. Cytomegalovirus, Epstein-Barr virus and varicella zoster virus infection in the first two years of life: a cohort study in Bradford, UK. BMC Infect Dis. 2017;17(220):1-18. 10.1186/s12879-017-2319-7 PMC5360071 28320319

[24] Pembrey L, Waiblinger D, Griffiths P, Wright J (2019). Age at cytomegalovirus, Epstein Barr virus and varicella zoster virus infection and risk of atopy: the born in Bradford cohort, UK. Pediatr Allergy Immunol.

[25] Deverill, C. *et al.* Health Survey for England 2002: The Health of Children and Young People. Methodology & Documentation. (The Stationary Office, 2002).

[26] Goscé L, Winter JR, Taylor GS, Lewis JEA, Stagg HR (2019). Modelling the dynamics of EBV transmission to inform a vaccine target product profile and future vaccination strategy. Sci Rep.

[27] Office for National Statistics (2018). Estimates of the population for the UK, England and Wales, Scotland and Northern Ireland.

[28] Office for National Statistics. Census boundary data [United Kingdom]. https://census.ukdataservice.ac.uk/get-data/boundary-data.aspx (2011). Accessed 16 Jan 2018.

[29] UK Data Service (2001). Census aggregate data.

[30] UKB : Data-Field 23054. https://biobank.ndph.ox.ac.uk/showcase/field.cgi?id=23054. Accessed 11 Oct 2020.

[31] Vyse AJ, Hesketh LM, Pebody RG (2009). The burden of infection with cytomegalovirus in England and Wales: how many women are infected in pregnancy?. Epidemiol Infect.

